# Population health trends and disease profile in Somalia 1990–2019, and projection to 2030: will the country achieve sustainable development goals 2 and 3?

**DOI:** 10.1186/s12889-022-14960-6

**Published:** 2023-01-10

**Authors:** Joana Morrison, Sk Md Mamunur Rahman Malik

**Affiliations:** 1 WHO Somalia, Carrer Sant Elies 22, 5-4, 08006 Barcelona, Spain; 2 WHO Representative & Head of Mission, World Health Organisation Country Office Mogadishu, Mogadishu, Somalia

**Keywords:** Somalia, Maternal mortality, Infant mortality, Disability-adjusted life years, Global burden of disease

## Abstract

**Objectives:**

This study aims to evaluate whether Somalia will reach Sustainable Development Goals 2 and 3 by 2030 and what the country requires to advance closer to these objectives.

**Setting:** Somalia.

**Participants:**

We carried out analyses of secondary data obtained from the following open-access databases: Global Burden of Disease 2019 study; United Nations (UN) Department of Economic and Social Affairs Population Division; World Bank World Development Indicators; United Nations Children’s Fund (UNICEF); UNICEF/World Health Organisation (WHO)/World Bank Joint Child Malnutrition Estimates; and UN Interagency Group for Child Mortality Estimation (UN IGME), disaggregated by sex.

**Primary outcome measures**: stillbirth, neonatal, infant, under-five, maternal and child mortality; under-five malnutrition; life expectancy; health-adjusted life expectancy; age-standardised all-cause mortality; age-standardised cause-specific mortality for the leading causes of death; disability-adjusted life years. **Secondary outcome measures**: vitamin A coverage; stunting, overweight in children under 5; top risk factors contributing to cause-specific mortality.

**Results:**

life expectancy in Somalia will increase to 65.42 years (95% UI 62.30–68.54) for females and 58.54 years (95% UI 54.89–62.19) for males in 2030. Stunting will continue to decline to 25.2% (90% UI 13.9–39.5%), and the under-five mortality rate will drop to 85.9 per 1000 live births (90% UI 22.0–228.1 per 1000 live births) for females and 96.4 per 1000 live births (90% UI 24.8–255.3 per 1000 live births) for males in 2030. This study’s analyses predict that the maternal mortality ratio in Somalia will decline to 696.42 deaths per 100,000 live births in 2030.

**Conclusions:**

there has been progress towards SDG targets in Somalia since 1990. To achieve these, Somalia requires greater health improvements than observed between 1990 and 2019.

**Supplementary Information:**

The online version contains supplementary material available at 10.1186/s12889-022-14960-6.

## Background

Somalia has some of the lowest-ranked health indicators worldwide [1]. Several decades of civil war have diminished Somalia’s health system and displaced 2.6 million people within the country [2, 3]. The ongoing flooding, droughts, locust plagues, and conflict in Somalia have caused sustained food crises and, ultimately, famines during 2010–2012 [4]. The b﻿urden of malnutrition is a considerable health concern in Somalia and jeopardises the growth and development of children and young people [5]. Sustainable Development Goal–2 (SDG) aims to ‘end hunger, achieve food security and improved nutrition and promote sustainable agriculture by 2030’ [6].﻿

In low- and middle-income countries, most maternal and infant deaths are preventable [7]. Over half of maternal deaths worldwide occur in sub-Saharan Africa, and the maternal mortality ratio in Somalia is one of the highest worldwide [8]. SDG-3 aims to ‘ensure healthy lives and promote well-being for all at all ages by 2030’ [9]. The SDGs establish ‘to leave no one behind’ as a core principle [10]. However, current Somali health and nutrition rankings do not bode well for progress towards reaching SDG 2–3 targets [11].

Good quality, up-to-date data are necessary for monitoring health outcomes, assessing progress in achieving SDGs and to inform policy [12]. This requirement is even more true in countries with fragile and fragmented health systems, such as Somalia, which has been in a protracted state of conflict for the last 30 years [4]. However, there is a sparseness of health information and evidence in Somalia [13]. Its Federal Ministry of Health (FMoH) collaborated with the country’s WHO regional office to develop a five-year strategic health plan (2021–2025). It covers a broad range of health concerns, such as communicable and non-communicable diseases and mother and child health. Together with the WHO country office in Somalia, this paper’s authors produced a report describing trends in population health and disease burden. The research evidence fed into the health plan and generated opportunities for further collaborative efforts between the FMoH and WHO. This study presents the report’s main findings.

This investigation describes trends in life expectancy and health-adjusted life expectancy (HALE) estimates, communicable and non-communicable diseases, mother and child health, injuries, and malnutrition in Somalia during 1990–2019. Projections for 2030 are presented to show whether Somalia will achieve SDG–2 and SDG–3 targets and where improvements exist and challenges persist.

## Methods

### Study design

To describe disease patterns in Somalia, we produce trends for 1990–2019 and calculate forecasts for 2020–2030 to appraise whether Somalia will reach SDG2 and SDG3 goals.

### Study variables

The study’s primary outcome measures are maternal mortality ratio (MMR), stillbirth rate (SBR), neonatal mortality rate (NMR), infant mortality rate (IMR), and under-5 mortality rate. Female and male life expectancy, female, and male health-adjusted life expectancy, age-standardised all-cause mortality, age-standardised cause-specific mortality for the leading causes of death, and disability-adjusted life years (DALYs). The secondary outcome measures are vitamin A coverage among 12–23-month-olds, under-five stunting and overweight, and leading risk factors contributing to cause-specific mortality.

### Data sources

#### Under-5 malnutrition

This study includes the following variables to track malnutrition in Somalia: two-dose coverage of vitamin A for 6–59-month-old infants, stunting and overweight. We accessed data on vitamin A intake from the UNICEF global database through the UNICEF NutriDash system [13]. The modelled estimates provided by UNICEF are available for 2001–2018. We downloaded all other malnutrition data through the UNICEF/World Health Organisation (WHO)/World Bank Joint Child Malnutrition Estimates Database [14]. Data for stunting are available for 2000–2020. 

#### Maternal and child health

Estimates of neonatal, infant, and child mortality vary by source and method, making comparisons over time difficult. For consistency, we accessed child mortality data from the Group for Child Mortality Estimation (UN IGME) [15], an inter-agency collaboration between UNICEF, WHO, World Bank and the UN, because it provides harmonized comparable data. Data for stillbirth are available for 2000–2019, while other child mortality indicators are available for 1990–2019 [15].

We used MMR estimates for 1990—2019 provided by the Institute for Health Metrics and Evaluation (IHME) and the 2019 GBD study [].

#### Burden of disease

We obtained data from the GBD 2019 study data tool for the following indicators for 1990–2019: life expectancy, HALE, age-standardised all-cause mortality, age-standardised cause-specific mortality for the leading causes of death and leading risk factors for cause-specific mortality and DALYs [16].

### Data analysis

#### Descriptive analysis

We used Stata 17 software to clean datasets and analyse trends, absolute changes and percentage changes during the study period [17].

#### Predictive analyses

To assess whether Somalia will reach health and nutrition-related SDG aims and objectives by 2030, we calculated projections for 2030 using Stata 17 software [17]. For this, we produced 11-year linear predictions employing the mean annual change for female and male HALE, vitamin A coverage, and overweight. We used the yearly median change for variables with a heavy-tailed distribution to calculate linear predictions (crude birth and death rate, adolescent and total fertility, female and male life expectancy, stunting, MMR). We performed stochastic simulation for population and life expectancy variables for which uncertainty intervals are unavailable [17]. Dynamic forecasts extended the dataset’s period to include the prediction horizon and calculate uncertainty intervals (UI) [17]. For all forecast analyses, we compared actual values with the forecasted ones to validate the models and assess accuracy.

## Results

### SDG-2 malnutrition

The stunting rate in children under five declined during 2000–2020, as did the percentage of overweight children under five. The forecasts in this study show that rates will continue decreasing until 2030 (Table 1). The coverage of infants aged 6- to 59 months with two doses of vitamin A fluctuated between 2001 and 2020 [13]. Forecasts in this study show that coverage will reach 44% of infants in 2030.

**Table 1:** Mean annual change during 1990–2020 and 2030 estimates for Somalia’s population indicators

Indicator	2019 value	Mean/median annual change 1990–2020	Estimated value in 2030
Total population	15,893,000	2.62	19,196,000
Life expectancy, females (years)	61.29	0.37	65.42 (95% UI 62.30–68.54)
Life expectancy, males (years)	55.73	0.35	58.54 (95% UI 54.89–62.19)
Birth rate (per 1000 population)	41.59	−0.38	42.30 (95% UI 40.97–43.64)
Crude death rate (per 1000 population)	19.6	−0.27	10.00 (95% UI 8.97–11.04)
Fertility rate (births per woman)	5.98	−0.08	5.48 (95% UI 5.28–5.68)
Adolescent fertility rate (births per 1000 women aged 15–19 years)	95.20	−0.05	94.22 (95% UI 70.80–117.66)
Females HALE (years)	53.14	0.28	56.19 (95% UI 51.66–60.16)
Male HALE (years)	49.88	0.27	52.83 (95% UI 48.57–56.93)
Female age-standardized death rate (per 100,000 population)	1415.10	−18.96	1206.55 (95% UI 949.65–1629.94)
Male age-standardised death rate (per 100,000 population)	1893.98	−23.55	1634.95 (95% UI 1313.74–2079.52)
Stillbirth rate (per 1000 total births)	28.38	−0.45	23.42 (95% UI 13.75–38.00
Maternal mortality ratio (per 100,000 live births)	829	−22.41	696.42 (95% UI 590.09–802.75)
Neonatal mortality (per 1000 live births)	36.86	−0.27	33.8 (95% UI 9.37–89.95)
Infant mortality, females (per 1000 live births)	67.9	−1	56 (90% UI 20.84–129.72)
Infant mortality, males (per 1000 live births)	79.4	−1	66.13 (90% UI 25.10–227.60)
Under-5 mortality, females (per 1000 live births)	110.6	−2.9	85.9 (95% UI 22–228.1)
Under-5 mortality, males (per 1000 live births)	122.6	−2.2	96.4 (95% UI 24.8–255.3)
Child mortality rate (per 1000 children aged 5–14 years)	25.3	−0.19	23.3 (95% UI 18.15–29.92)
Stunting in children under 5 (%)	27.4	−0.2	25.2 (95% UI 13.9–39.5)
Overweight in children under 5 (%)	2.9	−0.08	2.1 (95% UI 1.04–6.05)

UI: uncertainty interval; HALE: health-adjusted life expectancy

Note: maternal mortality ratio latest data is for 2017

### SDG-3

#### Life expectancy

**Fig. 1:**
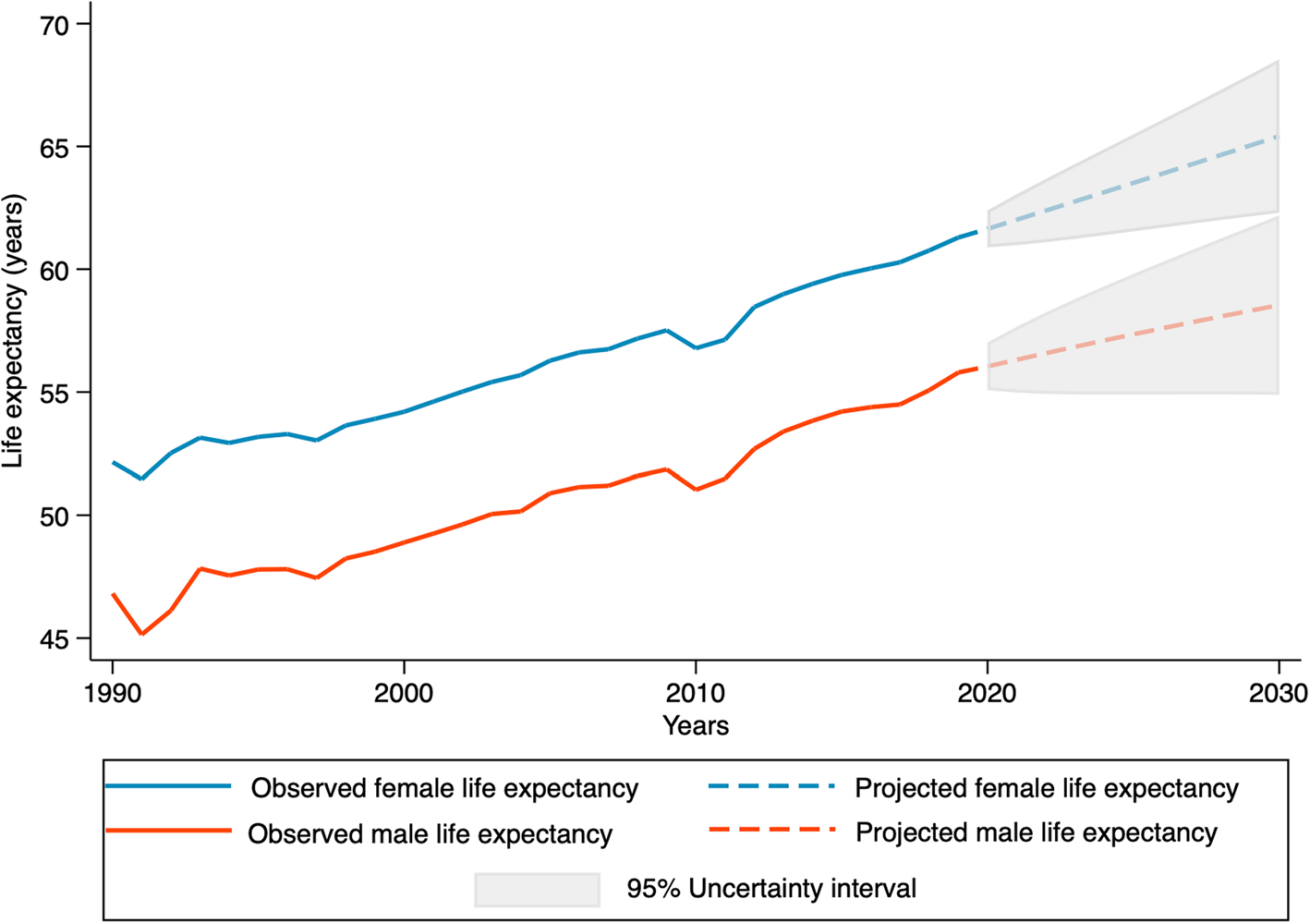
Female and male life expectancy at birth (years), Somalia 1990–2030. Observed values 1990–2019, estimated projections 2020–2030

Life expectancy at birth in Somalia saw an absolute increase of 12.6 years in females during 1990–2020 and a similar gain in males (12.2 years) (Fig. 1). Female and male HALE in Somalia rose by 8 years between 1990 and 2019 (see Table 1).

#### Maternal and child health

The maternal mortality ratio in Somalia declined between 2000 and 2017 (Table 1) [15]. Somalia will not meet the SDG 3.1 target of ‘reducing the maternal mortality ratio to less than 70 per 100,000 live births’ [18]. The MMR decrease rate needs to be six times higher than the reductions observed during 2000–2017.

There has been a downward trend for stillbirth and neonatal mortality rates during 1990–2019, with an absolute decrease of eight points (Table 1). Somalia will not meet the SDG 3.2.2 target of reducing ‘neonatal mortality to below 12 per 1000 live births’ [9]. There needs to be an average annual decrease eight times higher during 2020–2030 than between 1990 and 2019. Infant mortality in Somalia for females and males fell from 2007 to 2019 (Table 1). Under-5 mortality decreased between 2007 and 2019 for females and males. Somalia will not meet the SDG 3.2.1 target of reducing under-5 mortality to below 25 per 1000 live births by 2030 [9]. To do so, Somalia needs to decrease its under-5 mortality at thrice the rate during 1990–2019.

#### Burden of disease

Female and male age-standardised death rates in Somalia declined between 1990 and 2019 and will continue to fall until 2030 (Table 1). The study’s predictions shown in Table 2 report that mortality and DALYs decreased between 1990 and 2019 and will continue to decline until 2030. The attributable rate as a percentage of mortality and DALYs has decreased for communicable, maternal, neonatal, and nutritional diseases and increased for non-communicable diseases and injuries. The most significant changes in rankings of attributable rates are for HIV/AIDS, ranked 92nd in 1990 and 14th in 2019, and leishmaniasis (ninth in 1990 and 66th in 2019). This study’s forecasts show that in 2030, the contribution of communicable, maternal, neonatal, and nutritional diseases to mortality and disability will decline. While efforts to meet SDG targets 3.3 and 3.4 are in place, Somalia will only reach these in part in 2030. The attributable rate of non-communicable diseases will continue growing and decreasing for injuries.

**Table 2:** Deaths and DALYs per 100,000 population and rate of attribution (per cent) by three leading causes of mortality and burden of disease, Somalia, 1990, 2019 and 2030

Cause	Indicator	1990	2019	2030
Communicable, maternal, neonatal and nutritional diseases	Age-standardised mortality per 100,000	1274.15 (1100.74–1475.97)	761.53 (606.7–964.46)	494.78 (395.08–670.05)
	Contribution to mortality, %	74.48 (70.99–77.66)	65.01 (62.15–68.03)	60.27 (57.99–63.4)
	Age-standardized DALYs per 100,000	66,988.65 (59,280.09–74,981.63)	35,597.37 (28,787.95–44,342.27)	23,763.08 (18,969.98–30,846.7)
	Contribution to DALYs, %	78.63 (75.15–81.96)	68.82 (65.86–71.87)	62.5 (60.82–64.46)
Non-communicable diseases	Age-standardised mortality per 100,000	826.36 (667.75–993.39)	738.4 (587.21–924.46)	748.90 (597.15–931.58)
	Contribution to mortality, %	18.73 (15.88–21.82)	26.53 (23.85–29.40)	35.13 (34.26–36.33)
	Age-standardized DALYs per 100,000	28,553.12 (23,872.29–33,815.03)	25,354.95 (20,965.72–31,036.19)	25,272.73 (21,032.98–31,170.62)
	Contribution to DALYs, %	15.14 (12.28–18.30)	23.26 (20.30–26.26)	30.95 (28.35–33.37)
Injuries	Age-standardised mortality per 100,000	152.78 (125.19–186.31)	119.1 (90.87–156.25)	130.41 (101.50–168.59)
	Contribution to mortality, %	6.79 (5.75–7.93)	8.47 (7.13–10.47)	7.09 (5.97–8.62)
	Age-standardized DALYs per 100,000	7669.6 (6366.97–9338.42)	5739.72 (4483.96–7345.7)	6300.88 (5044.30–7930.53)
	Contribution to DALYs, %	6.23 (7.53–5.15)	7.92 (6.77–9.54)	6.49 (5.46–7.79)

DALYs: disability-adjusted life years

Note: Values in parentheses are 95% uncertainty intervals

**Fig. 2:**
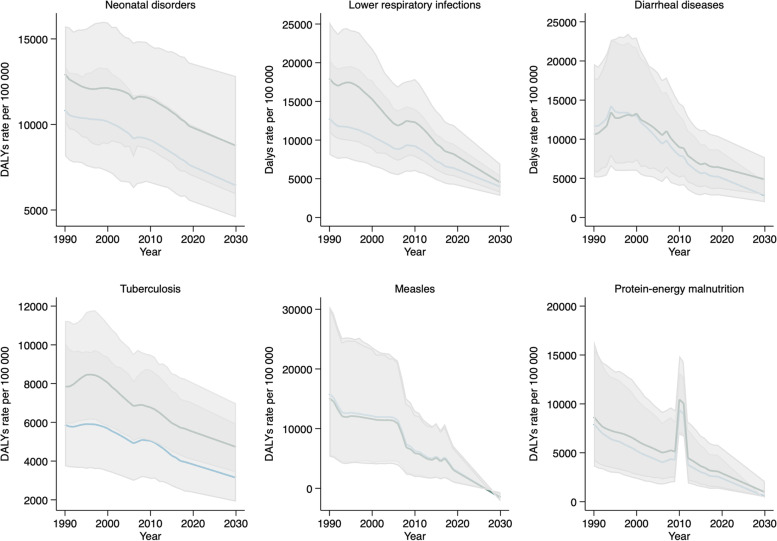
Leading causes of DALYs by sex, Somalia: observed values 1990–2019, estimated projections 2020–2030

As shown in Fig. 2, during 1990–2019, males had higher rates of neonatal disorders, respiratory infections and tuberculosis than females, and the leading causes of DALYs showed downward trends.

#### Risk factors

The five leading risk factors for mortality in Somalia are child and maternal malnutrition, air pollution, occupational risks and unsafe water, sanitation and handwashing, according to the GBD 2019 study [19]. Somalia’s top risk factors cause nutritional deficiencies and enteric, neurological, and musculoskeletal disorders and chronic respiratory diseases. Child and maternal malnutrition contribute to 49.57% of mortality, the leading risk factor for communicable, maternal, neonatal, and nutritional diseases. For non-communicable conditions, the leading risk factor is high blood pressure, contributing to 10.97% of mortality (metabolic risk). For injuries, the top risk factors are occupational risks contributing to 18.03% of mortality (environmental/occupational hazard) [19].

**Fig. 3:**
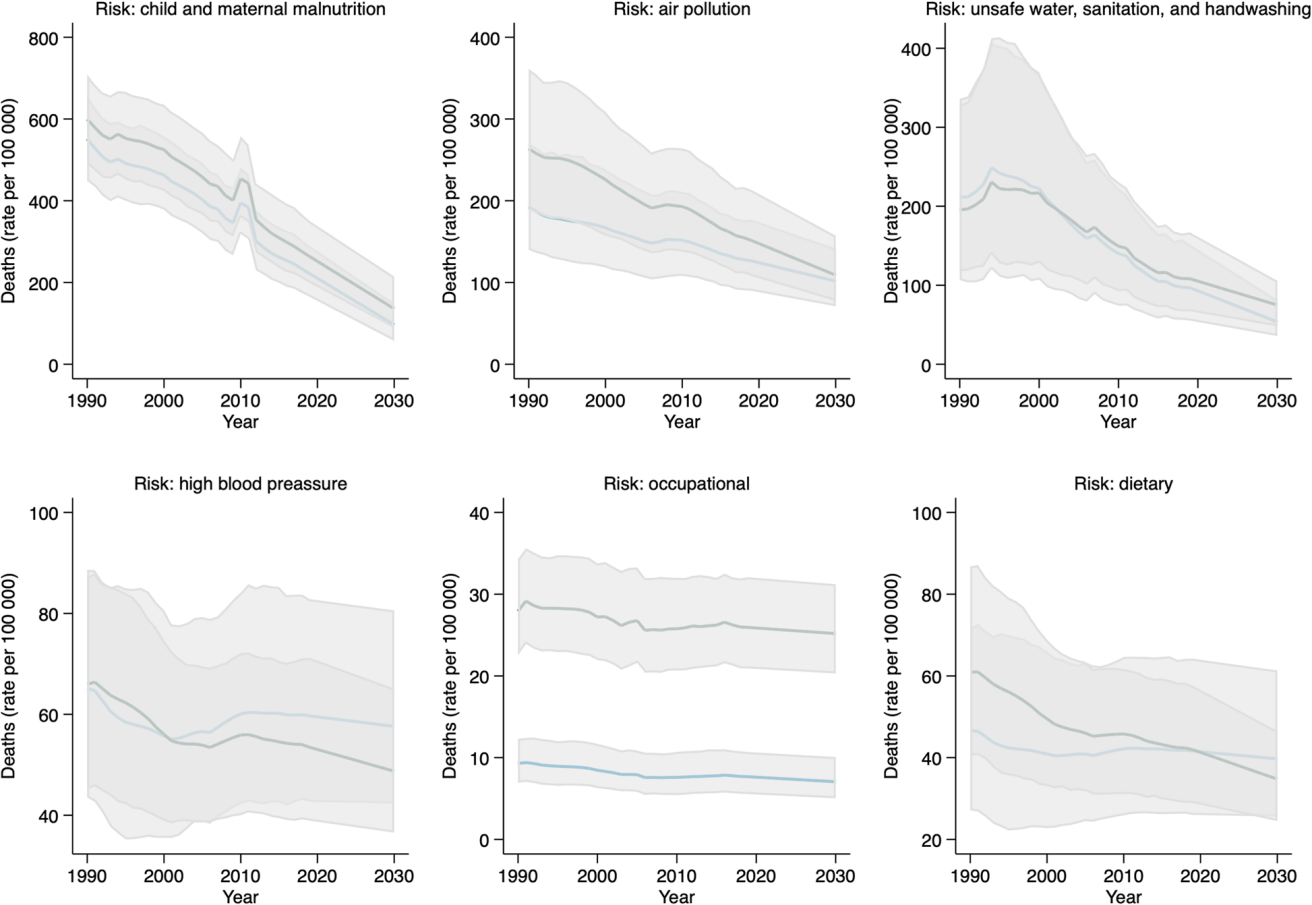
Trends in all-cause mortality by leading mortality risks in Somalia: observed values 1990–2019, estimated projections 2020–2030

Projections used in this study show that in 2030, child and maternal malnutrition will reach 96.63 per 100,000 population (95% UI 57.11–149.25) for females and 135.86 per 100,000 population (95% UI 88.37–214.31) for males. Female hypertension will reach 57.62 per 100,000 population (95% UI 42.34–80.58) in 2030, and male hypertension 48.73 (95% UI 36.58–65.10) for males (Fig. 3).

## Discussion

Life expectancy in Somalia has increased since 1990 (Table 1). Nevertheless, sustained mortality levels caused by communicable diseases and child and maternal mortality have possibly caused life expectancy to remain below the sub-Saharan average in 2020 (63.9 years for females and 60.3 for males) [19]. The relative stability experienced in Somalia since its recovery in 2012 with the establishment of a transitional federal government has driven an increase in healthcare, creating opportunities for universal health coverage (UHC) [20, 21]. However, Somalia’s health system remains weak; the UN reports that one-fifth of the population in Somalia still lack access to healthcare, which continues to pose severe challenges to the population’s health [22]. In response to the gaps in Somalia’s health care system, humanitarian actors have provided the following essential services [23]. The UNDP has worked towards reducing HIV/AIDS since 2004 by creating knowledge and awareness programs, increasing testing, and lobbying for HIV/AIDS legislation [24]. Managed by UNICEF, WHO and the United Nations Population Fund (UNFPA), Somalia’s Joint Health and Nutrition Programme (JHNP) has supported the institutional development of the Somali healthcare system and provided the Essential Package of Health Services (EPHS) [25]. The JHNP assists neonatal and reproductive health and supports child immunisations, nutrition and treatment of diseases in Somalia [3, 26]. It has possibly contributed to this study’s maternal and infant mortality reductions [26]. Data from MICS 2006 and the SHDS 2020 report that the percentage of deliveries assisted by health attendants and rates of pregnant women receiving antenatal care in Somalia increased between 2000 and 2019 [27, 28]. However, maternal health care remains lower than the sub-Saharan average and has yet to reach women equally. In Somalia, women of low income and living in rural areas have inadequate coverage [28].

UNICEF and WHO provided a parental awareness campaign for infant care-seeking, the Integrated Community Case Management [29]. SDHS data report that healthcare seeking for children under five with diarrhoea increased from 2.8% among rural children and 9% among urban children in 2006 to 52.2 and 59.9% in 2020 [28]. Care-seeking for children under age five with acute respiratory infections (ARI) in Somalia increased to 22.5% in 2019, up from 13% in 2006 [30]. The increased access to health care for children has contributed to the reduction in under-five mortality reported in this study.

Unvaccinated children in fragile or humanitarian settings are among the most vulnerable to disease outbreaks [31]. The vaccine initiative that UNICEF and WHO launched in 2013 in Somalia rolled out at least 1.3 million doses of the ‘five-in-one’ vaccine and provided a parental vaccine awareness campaign [32]. The vaccination initiative helped reduce under-five mortality rates in Somalia between 1990 and 2019. Nevertheless, child vaccination rates in Somalia remain low; only 42% of children aged 12–23 months are immunised against diphtheria, pertussis and tetanus (DPT) [33], 46% are vaccinated against measles [34] and 60.3% did not receive any vaccinations.

### Study limitations

The severe limitations and gaps in data availability in Somalia and the lack of access to individual-level health and undernutrition data are a limitation of this study. This is a crucial issue for describing and assessing improvements in mortality and disability. Weakened by decades of conflict and natural disasters, Somalia has yet to develop a wide-reaching health information system that allows the electronic collection and sharing of information [4]. Birth registration in Somalia is deficient; in 2019, only 6% of children under five were registered [35]. The latest MICS data for Somalia are for 2011 and are divided into the Northeast Zone and Somaliland [27]. Statistics provided by Africa UN (ECASTATS) offer essential data and information on SDGs [12]; however, there are very few available for Somalia and lack evidence strength. The GBD 2019 study provides forecasted data [19], and the estimations provided by GBD 2019 are well-established models; given the data paucity in Somalia, our study uses extrapolative models based on past trends, given the low data requirements [36, 37]. Linear forecasts enabled overcoming challenges of Somali health data. We analysed national-level estimates produced by international agencies using reliable data sources. These data allowed us to produce trends on many key health indicators to assess Somalia’s health development over the past three decades. Due to the variability of Somali data, we selected sufficient variables to ensure data quality.

## Conclusions

Somalia will not reach health and nutrition SDG targets by 2030. The moderate improvements observed in Somalia over the past three decades could act as a roadmap to accelerate progress and achieve SDG–2 and 3 aims.

In Somalia, maternal, infant, and under-five mortality remain a significant health challenge. A modest decline in maternal, infant and under-five mortality has contributed to female and male life expectancy improvements over the past thirty years. This trend is forecasted to continue in Somalia. Nevertheless, Somalia’s maternal and infant mortality rates remain high and highlights the need for measures to increase prevention among vulnerable populations. Addressing malnutrition in Somalia and reducing child stunting remains essential.

Somalia is still in the early stages of the health transition. Non-communicable diseases’ contribution to mortality and disability will continue to increase. While communicable, maternal, neonatal, and nutritional diseases remain a major health concern.

## Supplementary Information

**Additional file 1:** **Figure S1.** Trends in leading mortality causes, Somalia. Observed values 1990-2019, estimated projections: 2020-2030.

**Additional file 2:** **Figure S2.** Infant mortality rate (per 1,000 live births), Somalia 1990-2019, projected estimations for 2020-2030.

**Additional file 3:** **Figure S3.** Female and male health adjusted life expectancy at birth (years), Somalia 1990-2030.

## Data Availability

The datasets used and analysed during the current study are available from the corresponding author upon reasonable request.
