# Supplementary material for: Population health trends and disease profile in Somalia 1990–2019, and projection to 2030: will the country achieve sustainable development goals 2 and 3?
Source: BMC Public Health. 2023 Jan 10;23:66. doi: 10.1186/s12889-022-14960-6 (PMC9832660; doi:10.1186/s12889-022-14960-6)
Supplement: Supplementary file 1 — Additional file 1: Figure S1. Trends in leading mortality causes, Somalia. Observed values 1990-2019, estimated projections: 2020-2030. [file 12889_2022_14960_MOESM1_ESM.docx]

**Trends in leading mortality causes, Somalia. Observed values 1990 – 2019, estimated projections: 2020 – 2030**


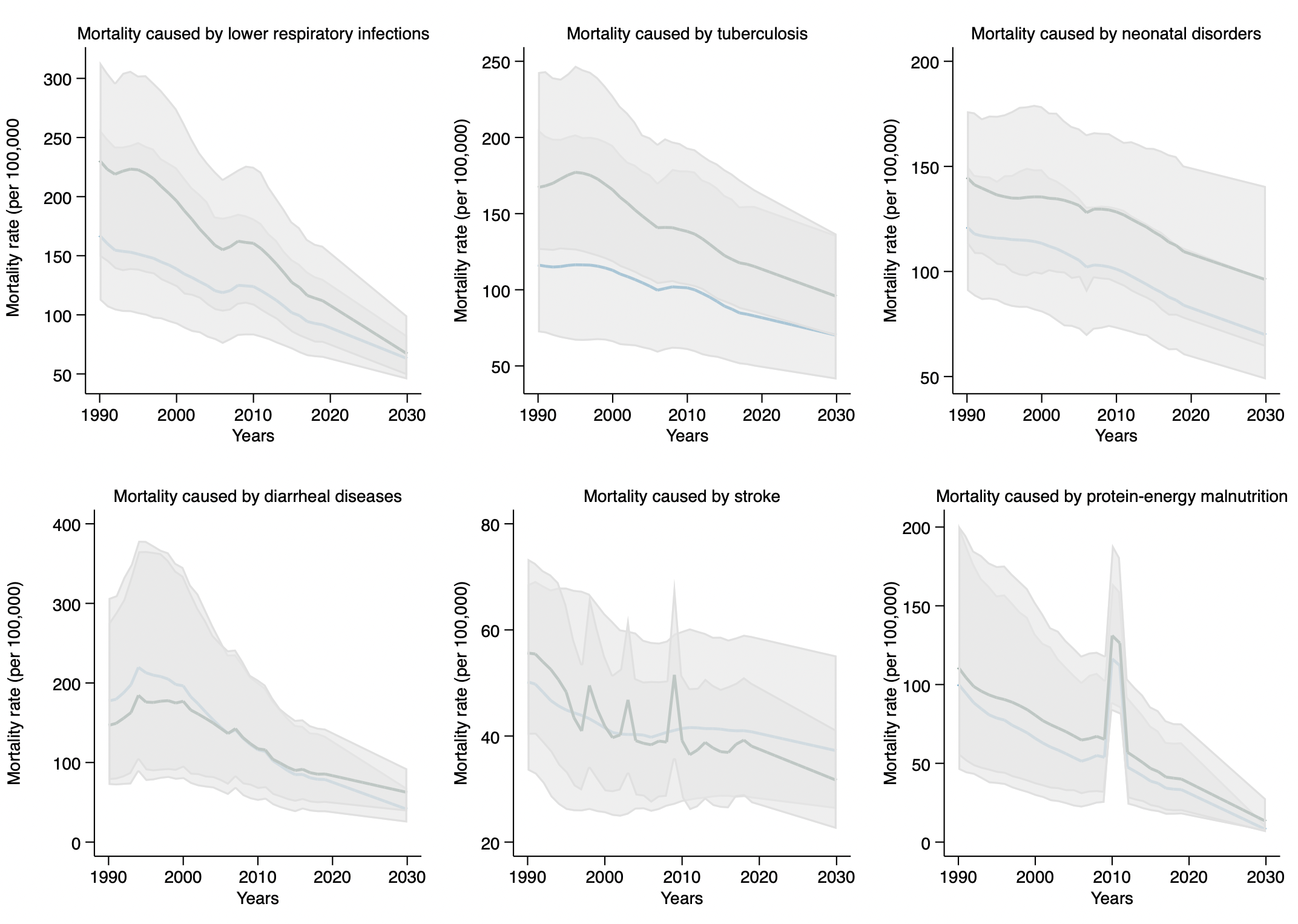


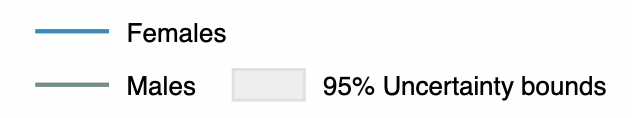


Note:

1. Lower and Upper bounds refer to the 95 percent uncertainty intervals for the estimates.
2. Observed values 1990 – 2019, estimated projections: 2020 – 2030
