# Supplementary material for: Population health trends and disease profile in Somalia 1990–2019, and projection to 2030: will the country achieve sustainable development goals 2 and 3?
Source: BMC Public Health. 2023 Jan 10;23:66. doi: 10.1186/s12889-022-14960-6 (PMC9832660; doi:10.1186/s12889-022-14960-6)
Supplement: Supplementary file 3 — Additional file 3: Figure S3. Female and male health adjusted life expectancy at birth (years), Somalia 1990-2030. [file 12889_2022_14960_MOESM3_ESM.docx]

**Female and male health adjusted life expectancy at birth (years), Somalia 1990 - 2030**

**
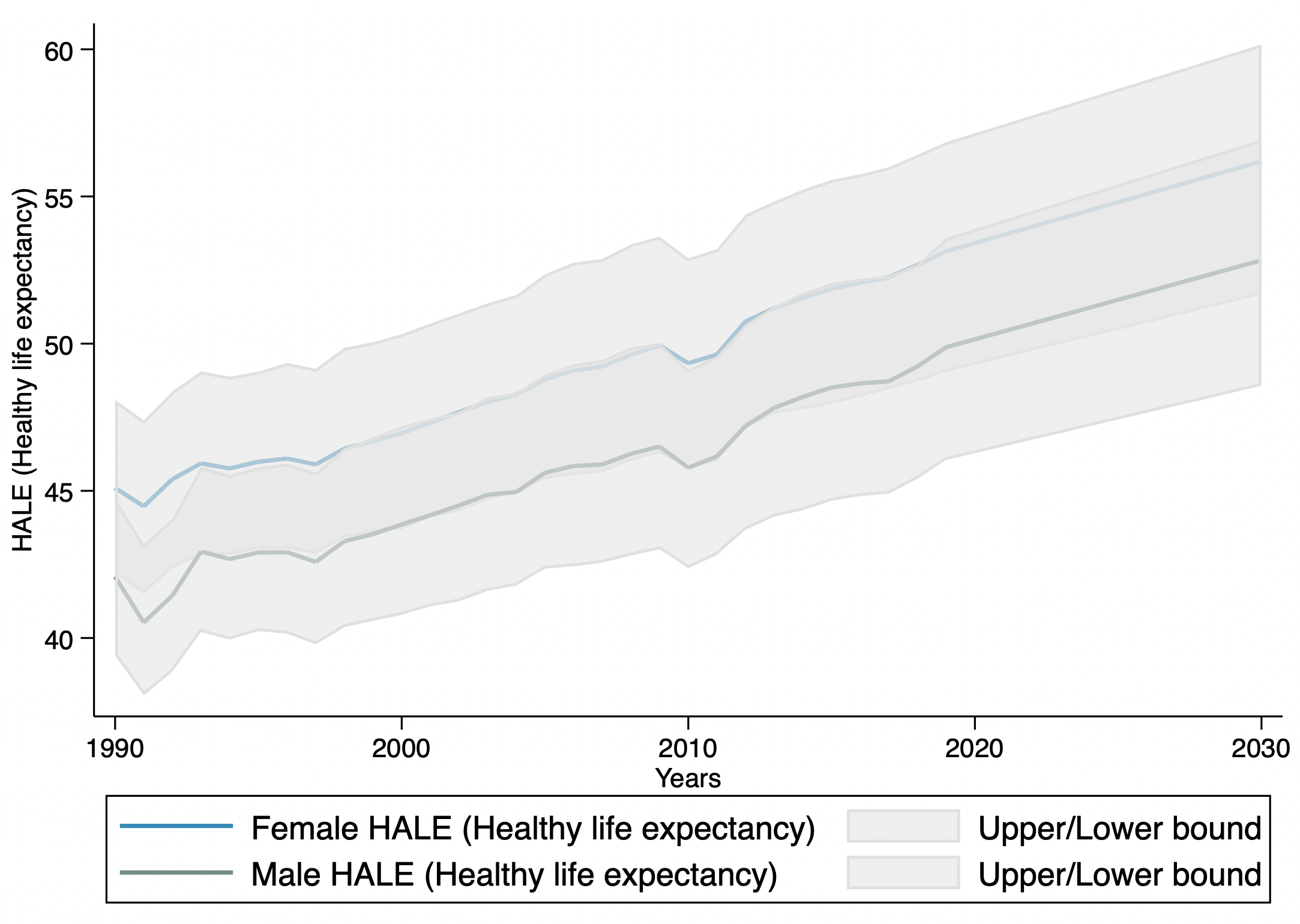
**

Note:

1. Lower and Upper bounds refer to the 90 percent uncertainty intervals for the estimates.
2. Observed values 1990 – 2019, estimated projections: 2020 – 2030
